# The rising cost of litigation for urological tumors in the Brazilian Public Health System: an analysis of e-NatJus technical notes (2020-2026)

**DOI:** 10.1590/S1677-5538.IBJU.2025.0516

**Published:** 2026-07-30

**Authors:** Fernando Korkes, Carlos Pedrotti, Flavio Tocci

**Affiliations:** 1 Faculdade de Medicina ABC - FMABC Disciplina de Urologia Santo André SP Brasil Disciplina de Urologia, Faculdade de Medicina ABC - FMABC, Santo André, SP, Brasil; 2 Hospital Israelita Albert Einstein Departamento de Urologia São Paulo SP Brasil Departamento de Urologia, Hospital Israelita Albert Einstein, São Paulo, SP, Brasil

To the editor,

Health litigation has become one of the major threats to the sustainability of the Brazilian Public Health System (SUS), and urological tumors occupy a prominent place in this phenomenon. We analyzed the technical notes issued by the Judiciary's Technical Support Centers (NatJus), made available through the National Council of Justice (CNJ) e-NatJus system, concerning malignant neoplasms of the genitourinary tract (ICD-10 C60–C68). Our aim was to assess the volume of demands, the most requested technologies, the rate of favorable opinions, and the evolution of the associated costs.

A total of 11,239 technical notes were identified for the C60–C68 group. The distribution is markedly concentrated: prostate cancer (C61) accounts for 6,218 notes (55.3%), followed by kidney cancer (C64) with 4,312 notes (38.4%) and bladder cancer (C67) with 567 notes (5.0%); the remaining topographies (C60, C62, C63, C65, C66, and C68) together represent less than 1.5% of the total. Prostate cancer is therefore the urological malignancy generating the greatest number of legal demands — a finding consistent with recent evidence that androgen receptor pathway inhibitors constitute an urgent public health problem in Brazil (1).

Using the Maximum Sale Price to the Government (MSGP) as a reference for the cost of the demanded technologies, a marked increase was observed over the study period. The aggregate value rose from BRL 450.9 thousand in 2020 to BRL 24.4 million in 2025 — an approximately 54-fold increase. The first half of 2026 alone already totals BRL 11.8 million, which, when projected to the full year, signals a level comparable to 2025 (approximately BRL 23.7 million). This growth is driven by two simultaneous vectors: the expansion in the number of demands (a 6.1-fold increase in notes between 2020 and 2025) and the rising unit cost of the requested technologies (the mean MSGP per priced note nearly doubled, from approximately BRL 8.8 thousand to BRL 18.5 thousand). Table-1 summarizes this evolution.

**Table 1:** e-NatJus technical notes for urological tumors (ICD C60–C68), aggregate cost by MSGP, and rate of favorable opinions, 2020–2026.

Year	Technical notes	Aggregate MSGP (BRL)*	Mean MSGP/note (BRL)	Favorable (%)
2020	392	450,854	8,840	74.2
2021	1,118	1,490,755	9,495	61.7
2022	1,529	1,430,066	9,408	60.3
2023	2,004	1,376,691	23,736	60.9
2024	2,353	5,297,857	16,504	50.4
2025	2,386	24,383,002	17,342	36.8
2026**	1,168	11,844,047	18,506	35.2

* MSGP summed only over notes with a registered price (a proxy for unit cost, not for the total cost of treatment).

** First half of 2026 only.

MSGP = Maximum Sale Price to the Government.

The profile of the requested technologies reflects the recent incorporation of high-cost drugs into oncological practice (1-3). In prostate cancer (C61), androgen receptor pathway inhibitors predominate — abiraterone (the most frequently demanded technology), enzalutamide, apalutamide, and darolutamide — along with radioligand therapies (lutetium-177 PSMA and radium-223) and olaparib; among procedures, robot-assisted radical prostatectomy, PSMA PET-CT, and radiotherapy stand out. In kidney cancer (C64), demands are concentrated on tyrosine kinase inhibitors (pazopanib, sunitinib, cabozantinib, axitinib, and lenvatinib) and immune checkpoint inhibitors (nivolumab, pembrolizumab, and ipilimumab). In bladder cancer (C67), immunotherapy (pembrolizumab, maintenance avelumab, atezolizumab, nivolumab, and durvalumab), the antibody-drug conjugate enfortumab vedotin, erdafitinib, and intravesical BCG are prominent (4).

Regarding technical merit, the C60–C68 group showed an overall rate of 50.7% favorable opinions. Notably, this rate declined over time: from 74.2% in 2020 to 36.8% in 2025 and 35.2% in the first half of 2026. In other words, as the volume and cost of demands grow, an increasing proportion of requests pertains to technologies that lack robust evidence or have not yet been incorporated into the SUS, thereby raising the proportion of unfavorable opinions.

These data align directly with the national urological literature. Korkes et al. estimated that litigation for prostate cancer alone may represent an annual burden of approximately BRL 783 million to the SUS, with a 30-fold increase in demands over four years and a concentration on androgen receptor pathway inhibitors, especially abiraterone (1). Similarly, this Journal has previously highlighted the rising costs of bladder cancer management in the public system and the need for structured public policies (2, 3). Our findings, drawn from an independent source (the e-NatJus repository of technical notes), reinforce and broaden this diagnosis for urological tumors as a whole.

We acknowledge several limitations: the MSGP is a reference unit price and underestimates the total cost of treatment, which depends on dose, duration, and line of therapy; furthermore, price capture within the notes improved throughout the period, making the absolute values conservative. Even so, the direction and magnitude (5) of the phenomenon are unequivocal.

In conclusion, litigation for urological tumors — led by prostate cancer and driven by high-cost oncology drugs — imposes a growing and potentially unsustainable financial burden on the SUS. The declining rate of favorable opinions suggests a progressive mismatch between judicial demand and the available scientific evidence. Coordinated action among health managers, the scientific community, the Judiciary, and patient advocacy groups is urgently needed, including the strengthening of health technology assessment processes and of the NatJus networks themselves, to ensure equitable, rational, and financially sustainable access to cancer treatment in Brazil.

The Authors
